# Efficacy and safety of diphereline 11.25 mg, microrelin 11.25 mg, and microrelin 3.75 mg in premenopausal patients with breast cancer: a non-inferiority randomized clinical trial

**DOI:** 10.1186/s12885-023-11614-7

**Published:** 2023-11-10

**Authors:** Safa Najafi, Maryam Ansari, Zahra Omidi, Asiie Olfatbakhsh, Shiva Moghadam, Esmat-o-Sadat Hashemi, Niki Najafi, Shahpar Haghighat

**Affiliations:** 1 Breast Cancer Research Center, Motamed Cancer Institute, ACECR, Tehran, Iran; 2 Faculty of life sciences and biotechnology, Shahid Beheshti University, Tehran, Iran

**Keywords:** Breast cancer, Diphereline, Microrelin, Gonadotropin-releasing hormone agonist, Iran

## Abstract

**Background:**

Diphereline is a Gonadotropin-Releasing Hormone agonist commonly used in patients with breast cancer. This study aimed to compare the efficacy and safety of one-month and three-month Microrelin injections produced by Homa Pharmed Company with three-month Diphereline injections manufactured by IPSEN, France.

**Methods:**

The study was a non-inferiority randomized clinical trial conducted between 2019 and 2023 on premenopausal women candidates for endocrine therapy. The participants were randomly assigned in blocks of six to one of three groups named A (Diphereline 11.25 mg), B (Microrelin 11.25 mg), and C (Microrelin 3.75 mg). The participants’ menopausal symptoms, estradiol, and FSH serum levels were recorded in three-month intervals for one year. The efficacy of each medication and its side effects were compared among the three groups by statistical analysis during the one-year follow-up.

**Results:**

The study included 133 patients with breast cancer. A decreasing trend in the serum levels of FSH and estradiol and an increasing trend of menopausal symptoms were recorded during the study. No specific side effects leading to drug disruption, hospitalization, or exclusion from the study were observed. Adjusting the effect of study group and time showed no significant changes in estradiol levels between groups B (p = 0.506) and C (p = 0.607) and group A. Also, serum FSH changes between groups B (p = 0.132) and C (p = 0.104) compared to group A were not significant. Moreover, the menopausal symptoms during the one-year follow-up did not significantly increase in group B (p = 0.108) and C (p = 0.113) compared to group A.

**Conclusions:**

It can be concluded that injections of both Microrelin 11.25 mg and 3.75 mg, produced by Homa Pharmed, Iran, are non-inferior in terms of effectiveness and incidence of menopausal symptoms compared to Diphereline, manufactured by IPSEN, France.

**Trial registration:**

IRCT.ir, IRCT20201227049847N1; Registered on 09/01/2021.

## Background

Breast cancer ranks among the most frequently detected cancers in women globally, with an approximate occurrence of 2.3 million new cases [1]. The prevalence and incidence rates of breast cancer vary significantly in different countries and regions [2]. In Iran, the number of breast cancer new cases was 17,467 in 2017 with an increasing trend since 2003 [3]. Identifying breast cancer prognostic factors and advances in treatment options have significantly improved breast cancer survival rates [4].

Estrogen is an important factor in breast cancer development and prognosis. In primary treatments of breast cancer, luteinizing hormone-releasing hormone agonist (LHRHa) can be used to reduce estrogen levels in patients with positive hormone receptors [5]. According to NCCN guidelines, adjuvant hormonal therapy is recommended for all patients with estrogen receptor (ER) and progesterone receptor (PR)-positive invasive cancer regardless of age or lymph node involvement [6]. Tamoxifen is the most commonly-used endocrine therapy drug, reducing the risk of recurrence by 47%. It is effective for both pre- and post-menopausal women [7]. In patients who are premenopausal at the time of diagnosis, the NCCN guideline recommends the use of Tamoxifen with or without ovarian suppression, which is possible with the use of LHRHa drugs [6]. These drugs inhibit luteinizing hormone (LH) and reduce the secretion of follicle stimulating hormone (FSH) from the pituitary gland, thereby reducing estrogen production [8].

The TEXT-SOFT trial showed that the combination of aromatase inhibitors such as Exemestane with ovarian inhibitors significantly reduces the chance of recurrence compared to Tamoxifen plus ovarian inhibitors [9].

Triptorelin is a GnRH analog which initially stimulates the pituitary gland. After continuous contact with its receptor, triptorelin can inhibit gonadotropin release by reducing the sensitivity or reducing the production of receptors [5]. Diphereline is a long-acting triptorelin. In Iran, Homa Pharmed Company has produced the Iranian version of Diphereline under the brand name of Microrelin, which is available in the market in a dose of 3.75 mg for 28 days. This pharmaceutical company also has another product with a dose of 11.25 mg, which is injected at three-month intervals. This study aims to compare Homa Pharmed Company’s one-month and three-month injectable Microrelin with the three-month Diphereline (IPSEN France), in terms of their effectiveness in inducing menopause (defined as cessation of menstruation and serum estradiol below 5–10 µg/ml) and managing menopausal symptoms and complications.

## Methods

This study is a non-inferiority RCT conducted at Motamed Cancer Research Institute (MCI) between 2019 and 2023. The primary objective was to examine the effectiveness, benefits, and side effects of a domestically produced medication called Microrelin, available in one-month and three-month formulations by Homa Pharmed pharmaceutical company, and compare them with a similar foreign brand, Diphereline 11.25 mg.

### Eligibility criteria

The study included premenopausal patients over the age of 18 with non-metastatic breast cancer and positive hormone receptor status who met the criteria for endocrine therapy All of the patients received chemotherapy regimen of AC4T4 (Adriamycin 60 mg/m2, Cyclophosphamide 600 mg/m2 – Docetaxel 75 mg/m2), and Tamoxifen for endocrine therapy. The exclusion criteria included the need to hysterectomy and oophorectomy, and participant’s unwillingness to continue participation.

### Method

Throughout the treatment, all patients were monitored for their menstrual status. Patients with inclusion criteria who did not experience cessation of menstruation were recruited in the study. Menopausal status in the three groups was evaluated by examining the trends in serum estradiol and FSH levels as well as the changes in menopausal symptoms over a one-year period.

Since all patients had normal menstruation periods before the start of the study, estradiol levels were not measured at baseline. In all three groups, serum estradiol and FSH levels were measured after 3, 6, 9, 12 months of drug administration. Menopausal symptoms and drug side effects were assessed using the Heinemann questionnaire at start of the study and the following 3, 6, 9, and 12 months.

At the outset of this study, we provided patients with sufficient explanation about the procedures involved and a written informed consent was obtained from all eligible participants. We emphasized that patients could withdraw from the study whenever they wanted. The prescribed drugs were free of charge. This study was approved by the Ethics Committee of Motamed Cancer Research Institute (IR.ACECR.IBCRC.REC.1397.003).

### Instruments

1. Demographic and clinical characteristics of the participants were registered in a checklist.
2. Serum estradiol and FSH titers were obtained from laboratory reports and recorded.
3. The severity of menopause-related complaints was assessed using the Menopause Rating Scale (MRS) designed by Heinemann et al. in 2003 [10]. The Persian version of this questionnaire have been localized and validated by Makvandi et al. by a Cronbach’s alpha coefficient above 0.7 [11]. MRS consists of 11 items in three subscales: somatic (4 items), psychological (4 items), and urogenital (3 items). The severity of complaint in each item is measured by a five-point Likert scale varying from severity 0 (no complaints) to severity 4 (very severe symptoms). Total and subscale scores are calculated by adding the scores of each item. Higher scores indicate more severity of menopausal symptoms. Any unexpected side effects were recorded by an oncologist in patients’ document.

### Randomization

**Fig. 1:**
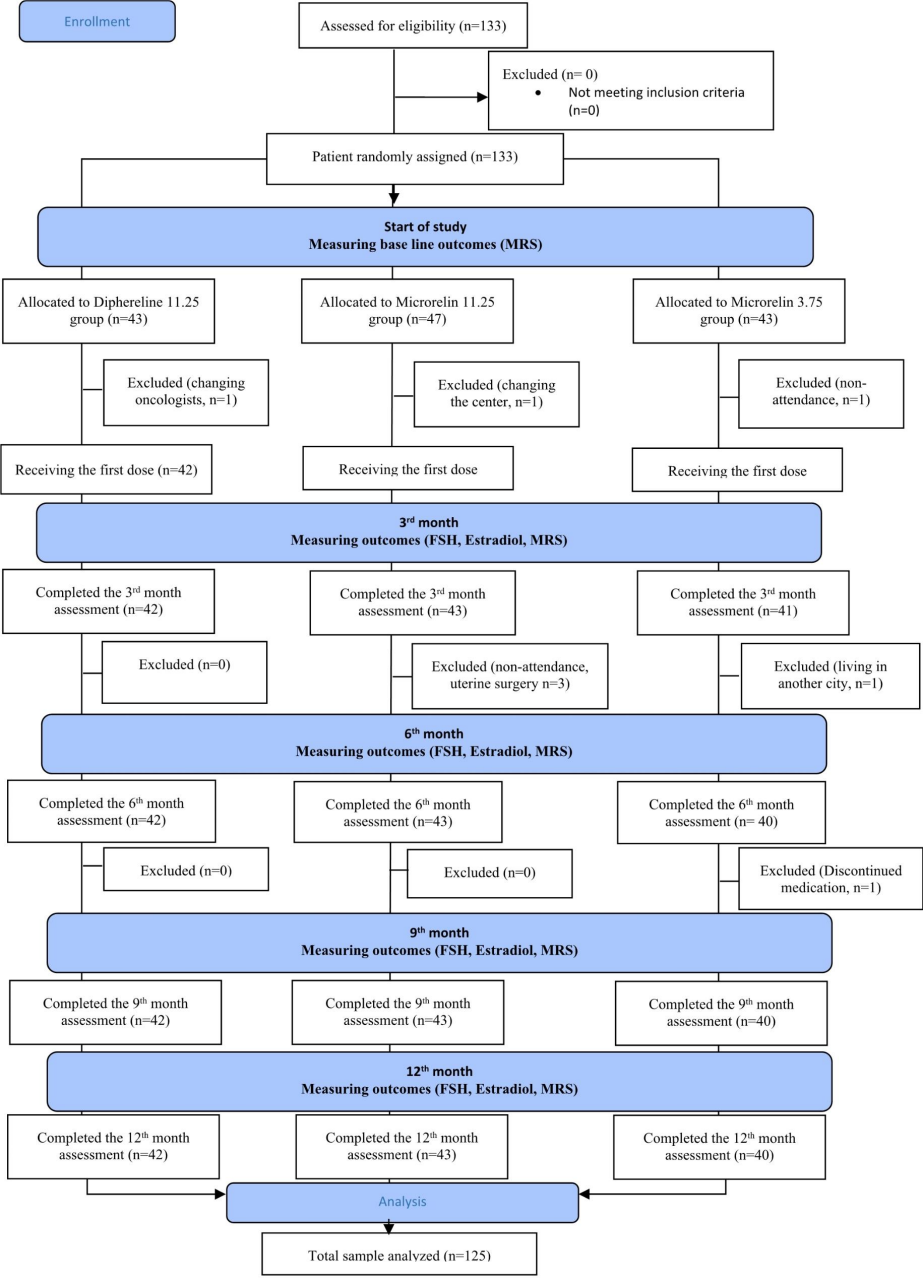
Consort diagram: recruitment, eligibility screening, randomization, follow-up, and analysis

Recruited patients were randomly assigned to one of the three treatment groups using a block randomization with a block size of six. A statistician created the blocks in sets of six using Excel software, resulting in 35 sets of different states (e.g., BBCAAC, BCAACB, CCAABB). The letters A, B, and C represented each of the three intervention groups, and each set of six random codes was placed in a sealed envelope. In recruitment phase, an envelope was randomly selected and the patients were assigned to one of the study groups based on the order of the letters (Fig. 1).

### Intervention groups

The intervention was one of the following three treatment methods:

Group A: Muscular injection of Diphereline 11.25 mg (produced by Ipsen France) in three-month intervals.

Group B: Muscular injection of Microrelin 11.25 mg (produced by Homa Pharmed Company) in three-month intervals.

Group C: Muscular injection of Microrelin 3.75 mg (produced by Homa Pharmed Company) in monthly intervals.

### Outcomes

The outcomes of the study consisted of serum estradiol and FSH levels and score of menopause symptoms every three months.

### Blinding description

In this study, a trained nurse in the research team recorded demographic and clinical characteristics, outcome results, and randomized allocation to different groups. In the two groups receiving three-month injections, patients and observers (oncologist and nurse) were blinded to the administered drug. Blindness was not possible in the group of monthly drug injection for patients, the nurse and the oncologist. The analyzer was completely blinded to patients’ allocation to the three groups up to the end of the study.

### Sample size

During the study, we tried to minimize loss to follow-up by close monitoring of the patients. The primary outcome under investigation was changes in serum estradiol and FSH levels over one year. In a similar study [12], which aimed to compare one-month and three-month injections of Goserelin, the ratio of the area under the curve of estrogen changes over time was 0.6 with a standard deviation of 0.09 after adjusting for the effect of baseline estrogen. As there were no similar studies on the drugs examined in our study, we determined the sample size considering an alpha error of 0.05, a beta error of 0.20, and a standard deviation of 0.1 for the mean changes in each arm. Assuming the difference of effect size up to 5% as an important clinical cut-off point in this non-inferiority trial, the sample size was taken as 44 patients per group. For comparing the three treatment groups and applying a coefficient of 1.4 in the sample size calculation formula, we needed 62 patients in each group. Considering a potential dropout rate of 15%, we planned to include 70 patients in each arm.

### Statistical analysis

Descriptive statistics were used to analyze the demographic and clinical characteristics of all participants in the three treatment groups. The frequency distribution of variables was compared among the groups to ensure proper randomization. The outcome variables were FSH and Estradiol levels and MRS scores which they had been quantitively measured 4 and 5 times during one year, respectively. There was only 8 missing data in three groups, which were excluded from analysis. They did not have normal distributions, so the Friedman test was used to examine changes of MRS scores, FSH and estradiol levels over time and the Kruskal-Wallis test was used to compare the mean difference of outcomes among the groups at each time point of measurement. Moreover, Generalized Estimation Equation (GEE) test was employed to investigate the interaction effect of time variable and treatment groups with the studied repeated measurement outcomes. Significance level of α error equal 5% was considered in the analysis.

## Results

In this study, we performed two interim analyses with 30% and 50% of the anticipated sample size. Both analyses confirmed the non-inferiority of drugs B and C compared to A. So, we achieved a final analysis on 133 patients of group A (n = 43), group B (n = 47), and group C (n = 43). A few participants were excluded as follows: one subject in group A (due to change of the oncologist), four in group B (due to loss to follow-up in MCI clinic), and three in group C (due to loss to follow-up and discontinuation of the drugs) (Fig. 1). The final assessment was performed on 125 breast cancer cases, including groups A (n = 42), B (n = 43), and C (n = 40).

Demographic and clinical characteristics of the participants in three arms are shown in Table 1. There was no significant difference in the demographic and clinical variables at the 5% error level, which confirms the appropriateness of the random allocation of the participants to the three study groups.

**Table 1:** Demographic and clinical characteristics of participants and comparison among the groups

Variable	Group A N (%)	Group B N (%)	Group C N (%)	P value^*^
**Stage of the disease**				0.784
I	11 (26.2)	12 (27.3)	11 (33.3)	
II	30 (71.4)	31 (70.5)	20 (60.6)	
III	1 (2.4)	1 (2.3)	2 (6.1)	
**Grade**				0.434
I	3 (9.1)	1 (2.6)	2 (5.7)	
II	24 (72.7)	31 (79.5)	31 (88.6)	
III	6 (18.2)	6 (15.4)	2 (5.7)	
II/III	0 (0)	1 (2.6)	0 (0)	
**Her2neu**				0.410
Negative	37 (86)	43 (89.6)	37 (94.9)	
Positive	6 (14)	5 (10.4)	2 (5.1)	
**Comorbidities**				0.640
No	35 (81.4)	42 (87.5)	34 (81)	
Yes	8 (18.6)	6 (12.5)	8 (19)	
**Pathology report**				0.887
IDC	24 (55.8)	27 (57.4)	25 (61)	
Others	19 (44.2)	20 (42.6)	16 (39)	
**Marital status**				0.981
Single	4 (9.3)	5 (10.4)	4 (9.5)	
Married	36 (83.7)	41 (85.4)	36 (85.7)	
Divorced/Widowed	3 (7)	2 (4.2)	2 (4.8)	
	**Mean (± SD)**	**Mean (± SD)**	**Mean (± SD)**	**P value**
Age	41.44 (5.01)	42.31 (5.29)	40.57 (7.07)	0.374^**^
BMI	27.36 (4.10)	27.38 (4.95)	27.49 (4.56)	0.989^**^
No. of gravidity	1.92 (1.11)	2.37 (1.35)	2.13 (1.32)	0.278^**^
Tumor size (cm)	2.37 (2.10)	2.19 (0.99)	2.36 (1.66)	0.877^**^
Ki67	23.21 (15.62)	31.28 (23.58)	28.38 (19.32)	0.162^**^
FSH_3	8.70 (9.21)	9.17 (7.70)	9.26 (8.10)	0.078^***^
Estradiol_3	13.28 (15.04)	12.28 (15.20)	7.45 (9.46)	0.089^***^
MRS_0	11.74 (8.44)	13.00 (8.35)	12.14 (6.91)	0.650^***^

*Chi-square test

** One-Way ANOVA test

***Kruskal-Wallis test

MRS: Menopause rating scale. Her2: Human epidermal growth factor receptor 2. IDC: Invasive ductal carcinoma. BMI: Body mass index. FSH: Follicle stimulating hormone

**Fig. 2:**
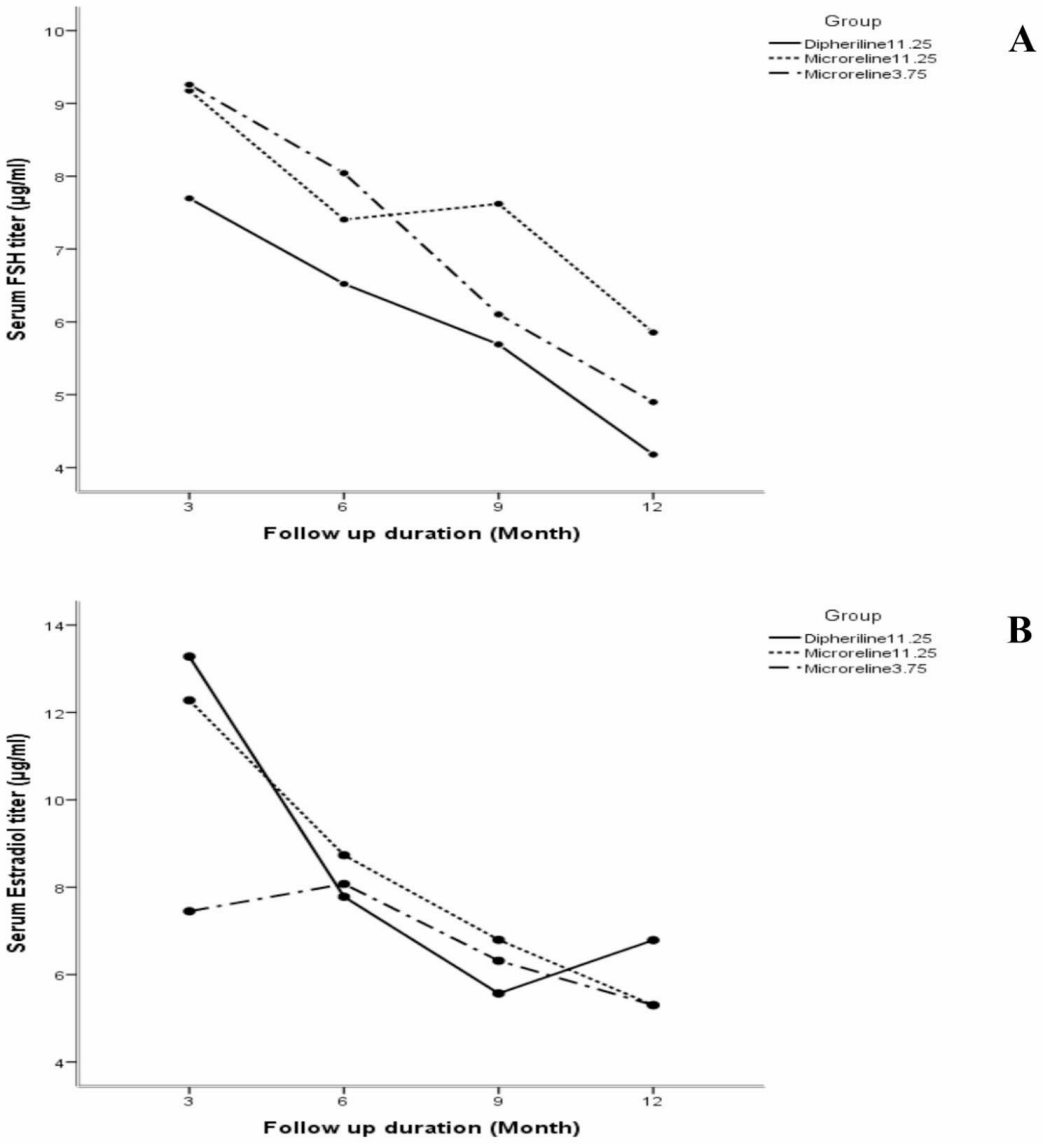
One-year changes in serum FSH **(A)** and estradiol **(B)** levels in the three groups

Table 2 shows that the serum level of FSH and estradiol in three arms had a decreasing trend during one-year period (Fig. 2). Changes in FSH serum level were significant in group B (p = 0.001) and group C (p < 0.001).

**Table 2:** Changes in FSH and estradiol during the three-month intervals in groups A, B and C

Variable	Group A	Group B	Group C	*P value* ^*b*^
	Mean (± SD)	Mean (± SD)	Mean (± SD)	
**FSH**				
3rd month	7.69(9.21)	9.17(7.70)	9.25(8.10)	0.078
6th month	6.52(7.36)	7.40(4.84)	8.04(6.03)	0.156
9th month	5.69(6.95)	7.62(4.27)	6.10(4.48)	0.006
12th month	4.18(3.25)	5.85(4.33)	4.90(3.45)	0.111
***P value*** ^***a***^	0.084	0.001	< 0.001	
**Estradiol**				
3rd month	13.28(15.04)	12.27(15.20)	7.45(9.46)	0.089
6th month	7.78(9.03)	8.73(7.35)	8.07(8.35)	0.351
9th month	5.57(8.04)	6.79(6.24)	6.32(6.08)	0.288
12th month	6.79(8.29)	5.30(5.67)	5.30(5.91)	0.896
***P value*** ^***a***^	0.001	0.025	0.312	

P^a^ Within-group comparison (Friedman Test)

P^b^ Between-group comparison (Kruskal-Wallis Test)

**Fig. 3:**
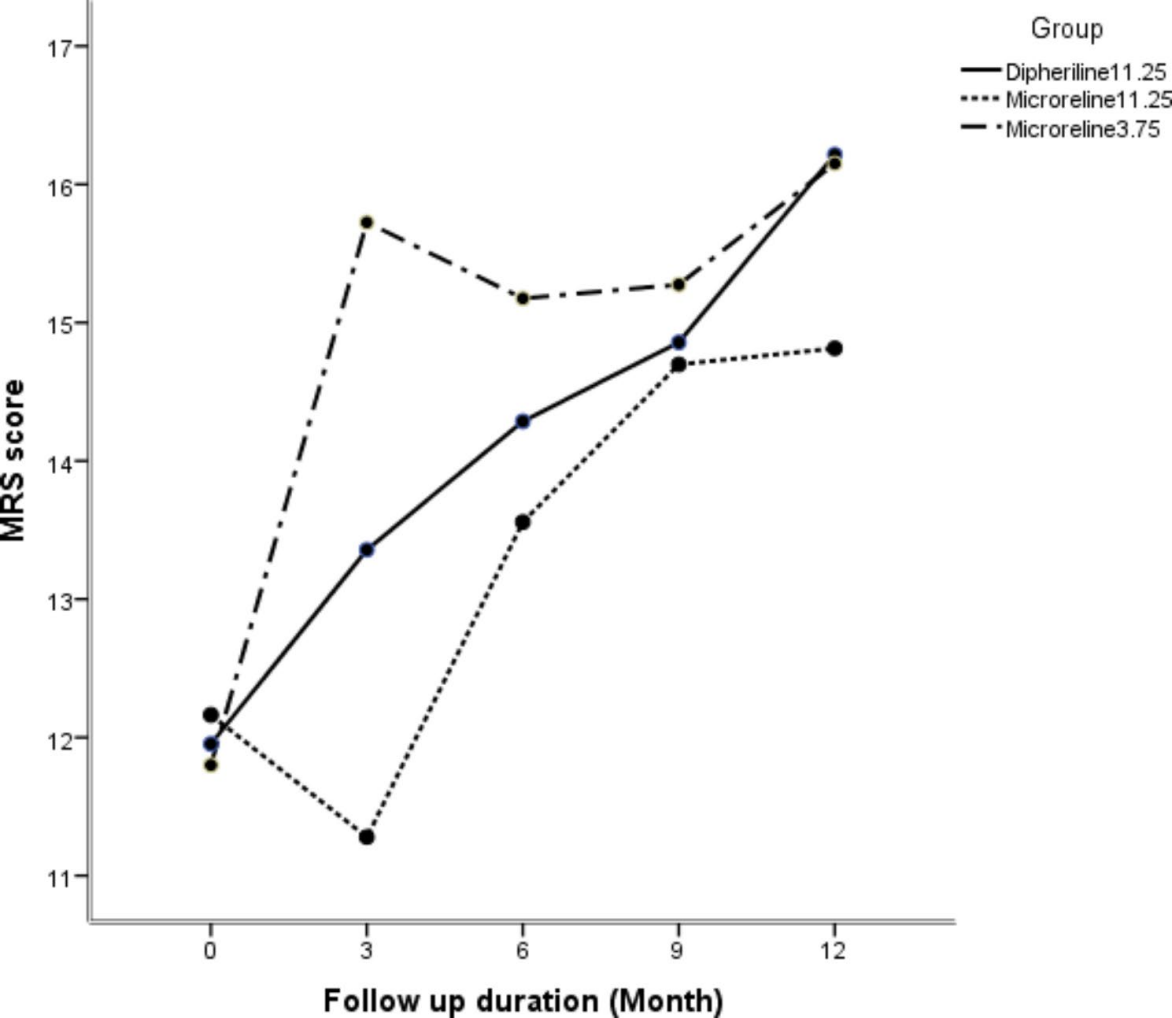
One-year changes in the mean score of MRS in the three groups of study

Changes in MRS and its subscales scores are summarized in Table 3. During the study, the total mean score increased significantly in groups A (p = 0.002), B (p = 0.001), and C (p = 0.003) (Fig. 3). The changes of somatic subscale score were significant in group A and C (p < 0.001, p = 0.001, respectively). Urogenital symptoms had increasing significant trend in three groups during one-year follow up (p < 0.001).

**Table 3:** One-year mean changes of MRS score and its subscale within and between groups

Variable	Group A	Group B	Group C	*P value* ^*b*^
	Mean (± SD)	Mean (± SD)	Mean (± SD)	
**Total**				
Start	11.95(8.43)	12.16(7.24)	11.80(6.77)	0.879
3rd month	13.36(7.34)	11.28(7.54)	15.72(6.96)	0.020
6th month	14.29(8.26)	13.56(7.13)	15.18(6.98)	0.543
9th month	14.56(8.50)	14.70(8.35)	15.28(7.46)	0.895
12th month	16.21(8.44)	14.81(8.37)	16.15(8.45)	0.711
***P-value*** ^***a***^	0.002	0.001	0.003	
**Somatic**				
Start	4.17(3.45)	5.00(3.33)	4.53(3.11)	0.390
3rd month	5.29(2.71)	4.77(3.10)	6.53(3.00)	0.038
6th month	5.74(3.04)	5.86(3.140	6.40(3.20)	0.497
9th month	6.43(3.17)	5.81(3.39)	6.48(3.05)	0.739
12th month	6.71(3.24)	6.05(3.48)	6.98(3.22)	0.491
***P-value*** ^***a***^	< 0.001	0.109	0.001	
***Psychological***				
Start	4.93(4.05)	4.30(3.66)	5.18(3.46)	0.483
3rd month	5.12(3.81)	3.84(3.98)	6.25(4.09)	0.013
6th month	5.45(4.14)	4.60(3.26)	5.83(3.64)	0.358
9th month	5.10(4.05)	4.95(3.72)	5.80(4.13)	0.615
12th month	5.71(3.68)	4.88(3.49)	5.88(4.13)	0.494
***P-value*** ^***a***^	0.702	0.087	0.371	
**Urogenital**				
Start	2.86(2.53)	2.86(2.35)	2.10(2.01)	0.286
3rd month	2.95(2.38)	2.79(2.32)	2.98(2.17)	0.912
6th month	3.10(2.45)	3.09(2.14)	2.95(2.50)	0.846
9th month	5.10(4.05)	4.95(3.72)	5.80(4.13)	0.615
12th month	3.79(2.59)	3.88(2.88)	3.30(2.67)	0.561
***P-value*** ^***a***^	< 0.001	< 0.001	< 0.001	

P^a^ Within-groups comparison (Friedman Test)

P^b^ Between-groups comparison (Kruskal Wallis Test)

GEE was used to adjust the interaction effect of time and group variables in order to compare the effectiveness and side effects of the drugs among the three groups within the study period of one year (Table 4). The results of the GEE test showed that the changes in the serum levels of estradiol and FSH in the arms of Microrelin 11.25 and Microrelin 3.75 (manufactured by Homa Pharmed Company) were not significantly different compared to the similar drug (Diphereline manufactured by Ipsen France Company). Microrelin 3.75 reduced the mean level of estradiol up to 0.521 units per three months more than Diphereline. Meanwhile, a lesser decrease in the mean estradiol level by 0.66 units per three months was observed after injection of Microrelin 11.25 compared to Diphereline.

Injection of Microrelin 3.75 and Microrelin 11.25 reduced the mean FSH level more than Diphereline 11.25 by up to 0.88 and 1.25 units per three months, respectively.

According to the findings (Table 2), all three drugs increased the total score of MRS over one year, but as shown in Table 4, a lower increase was achieved in the total score of MRS in Microrelin 3.75 and Microrelin 11.25 mg compared to Diphereline 11.25 mg by 0.24 and 0.22 units over three months, respectively.

**Table 4:** Interaction of time and group effect with the total MRS score, serum estradiol and FSH levels

Variable	Group/Time	β	SE	Wald Chi-Square	df	P value
**Estradiol**	Micro 11.25/Time	0.657	0.99	0.443	1	0.506
	Micro 3.75/Time	-0.521	1.01	0.265	1	0.607
	Dipher 11.25	0				
**FSH**	Micro 11.25/Time	-1.246	0.83	2.265	1	0.132
	Micro 3.75/Time	-0.878	0.54	2.651	1	0.104
	Dipher 11.25	0				
**MRS**	Micro 11.25/Time	-0.221	0.14	2.580	1	0.108
	Micro 3.75/Time	-0.236	0.15	2.509	1	0.113
	Dipher 11.25	0				

*Adjusted for time and group

## Discussion

Given the rising trend of breast cancer in the world and in Iran and the ensuing widespread use of GnRH agonists by these patients, physicians and patients must have access to products with the least side effects and the greatest effectiveness. This non-inferiority RCT aimed to compare the effectiveness of two injectable medications, i.e., one-month and three-month Microrelin (Homa Pharmed Company) and three-month Diphereline (IPSEN France), in terms of estrogen and FSH reduction as well as the occurrence of menopause complications and symptoms. Examining the changes in the variables over one year showed that the variations in estradiol and FSH levels as well as the drug side effects and symptoms of menopause did not differ significantly in Microrelin 3.75 and 11.25 mg groups compared to the Diphereline group and they can be considered in Iranian patients’ treatment.

During the study period of one year, a decreasing trend was observed in serum FSH and estradiol levels in all three treatment arms. Bellet et al. examined 116 premenopausal patients with breast cancer. After the patients received triptorelin, the estradiol level decreased by more than 95% in months 3, 6, and 12 compared to the outset of the study [13]. Another study compared the effects of tamoxifen + triptorelin (GnRH agonist) with letrozole + triptorelin on 81 premenopausal patients with breast cancer. Their findings showed that the level of estradiol and FSH significantly declined six months after the intervention in both groups. Triptorelin + letrozole had reduced ovarian function to a greater extent than the other group [14]. This result consists on the efficacy of three dugs during one-year period of study. Recruited patients consisted of premenopausal women with regular monthly menstrual cycles and their menstrual hormones might be affected by different factors such as environmental, nutritional, psychological, etc. in each cycle. In order to decrease the costs of lab tests, the baseline value of FSH and steroid were not measured, while menopausal symptoms were recorded by MRS questionnaire at start of study. Even though, the baseline value may not effect on the variation of serum FSH and steroid in different drug groups during the study, but its measurement can be considered in future researches.

Some factors such as the type of chemotherapy regimen, estrogen agonist drugs, and the time of GnRH agonist administration (which can be simultaneously with or after the chemotherapy drug) may affect the performance of the drug and its effectiveness in reducing ovarian function. Still, despite the presence of other factors influencing the effectiveness of cancer treatment, the results of the present study confirmed the effect of all three drugs on FSH and estradiol reduction. Only the level of FSH in the 9th month was slightly lower in the Diphereline group than in the other two groups (P = 0.006), although this finding did not have a marked effect on the overall results of the study, i.e. the comparison of the efficacy and side effects of the three studied drugs over one year. Investigating potential influencing and confounding factors in the effectiveness of GnRH agonists certainly requires independent, extensive, and controlled studies. The effectiveness of the three drugs over time was compared using linear regression and by adjusting the effect of group and time. The results showed that the mean serum estradiol and FSH level reduced by 0.521 and 0.88 units more with Microrelin 3.75 mg injection over three months compared to Diphereline. The injection of Microrelin 11.25 mg also reduced FSH level by 1.25 units more than Diphereline, but it decreased the estradiol level less by 0.66 units. Since the mentioned variations were not significant at the 5% level and showed a very small difference in the one-year efficacy trend curve, the lack of superiority in the efficacy of Diphereline compared to the two Microrelin drugs manufactured in Iran can be confirmed.

Therefore, to save foreign exchange costs, reduce the treatment costs of breast cancer, and due to the cost-effectiveness of the two similar drugs manufactured by Homa Pharmed Company, the use of the Iranian GnRH agonist is recommended. Note that GnRH agonists are also administered in the treatment of other diseases such as abnormal uterine bleeding, premature puberty, and prostate, uterine, and ovarian cancers; therefore, the approval of domestic drugs can save costs and develop domestic pharmaceutical industries.

The drug efficacy diagram (Fig. 2) in the 12th month of follow-up demonstrated a slight elevation in estradiol level in the Diphereline group compared to the other two groups. It may seem that the long-term effect of the foreign Diphereline is less than that of similar Iranian drugs; however, it is crucial to consider the influence of other factors such as age distribution, hormonal reactions of different people, possible underlying diseases, mental status changes, and the effect of concurrent cancer treatments. The decision-making process for selecting the most suitable adjuvant endocrine therapy should always incorporate the evaluation of treatment toxicities and involve thorough discussions with patients, taking into consideration their preferences and comorbidities, even during the adjuvant treatment [15]. Even though an appropriate randomization, close follow up and limiting the loss of follow up decreased the methological biases, but designing an study with a longer follow-up period that takes into account several influencing factors may help clarify the real efficacy of the drug with greater power.

The findings of this study showed that during the course of the study, the mean score of total menopausal symptoms increased significantly in all three groups, which demonstrates the effect of all three drugs on the induction of menopause and its symptoms. Still, in none of the three groups was there any specific side effect that led to drug discontinuation, hospitalization, or exclusion from the study –an observation that emphasizes the safety of the two domestically-manufactured drugs. Menopause involves physical symptoms such as hot flashes, psychological symptoms such as depression, cognitive problems, and urinary-reproductive symptoms such as vaginal dryness [16]. An in-depth examination of the subscales of the MRS showed an increase in the mean score of the *somatic* subscale over time in all three groups, and this rise was significant in the Diphereline and Microrelin 3.75 mg groups. Nonetheless, the *psychological* sub-scale did not show a significant difference in any of the groups. This finding emphasizes the fact that hot flashes, palpitations, sleep disorders, and muscle and joint pain are the predominant symptoms of menopause, which manifest themselves more than psychological symptoms such as depression, anxiety, anger, and forgetfulness due to hormonal changes. A study on the effect of ovarian function suppression (mainly with triptorelin along with tamoxifen or exemestane) on the cognitive function of breast cancer patients showed that ovarian function suppression and endocrine therapy did not have a significant clinical effect on the women’s cognitive function [17]. Francis et al. studied women with breast cancer who received GnRH agonists (mainly triptorelin and, occasionally, diphereline 3.75 mg) along with tamoxifen. In these women, compared to women receiving tamoxifen, complications such as hot flashes (93.4% vs. 79.8%), sweating (61.8% vs. 48.3%), decreased libido (47.5% vs. 42.4%), vaginal dryness (49.8% vs. 41.8%) and musculoskeletal symptoms (75.1% vs. 69%) were more frequent –indicating the effect of GnRH agonists on suppressing ovarian function and its symptoms [18]. As in the TEXT and SOFT trials ovarian function suppression was achieved by same drug, patient-reported physical symptoms was because of different anti estrogen medicines [19], so it is better to suppress ovarian function use a drug that has fewer side effects in terms of causing physical and psychological symptoms. In the present study, the results of the GEE test revealed that the mean total score of menopausal complaints and symptoms increased 0.24 and 0.22 units less with the injection of Microrelin 3.75 and 11.25 mg compared to Diphereline over the three months. Therefore, it seems that the two drugs, i.e., Microrelin 3.75 and 11.25 mg, caused milder symptoms of menopause while having similar performance to Diphereline in inducing menopause. Although the difference between the three drugs was not significant, the two locally-manufactured drugs seem promising in terms of the quality of life of women.

This randomized clinical trial was achieved in a breast cancer center in Tehran which their patients were referred from different cities of Iran. It seems that hormonal effect of the studied three GnRH agonists drugs can be generalized to the breast cancers of other centers in this country. Studying the long-term effect of these drugs in future researches can provide valuable evidences for planning the breast cancer treatment protocol in premenopausal women.

## Conclusions

Based on the results of the study, it can be concluded that both Microrelin.

injections 11.25 mg and 3.75 mg, produced by Homa Pharmed Iran, are non-inferior in terms.

of effectiveness and incidence of menopausal symptoms compared to Diphereline manufactured by IPSEN France. Therefore, the use of domestically-produced drugs such as Microrelin injections could help save foreign exchange costs and reduce the medical expenses incurred by breast cancer patients without compromising their treatment outcomes or safety.

## Data Availability

The datasets used and analyzed during the current study are available from the corresponding author on reasonable request.

## Declarations

### Trial availability

This study was registered at the Iranian Registry of Clinical Trials (IRCT) on https://www.irct.iron09/01/2021. The registration code: IRCT20201227049847N1.

## Abbreviations

LHRHa: Luteinizing hormone-releasing hormone agonist
ER: Estrogen receptor
PR: progesterone receptor
FSH: Follicle stimulating hormone
LH: Luteinizing hormone
GnRH: Gonadotropin releasing hormone
